# Pre-control relationship of onchocercal skin disease with onchocercal infection in Guinea Savanna, Northern Nigeria

**DOI:** 10.1371/journal.pntd.0005489

**Published:** 2017-03-29

**Authors:** Michele E. Murdoch, Ian E. Murdoch, Jennifer Evans, Haliru Yahaya, Ngozi Njepuome, Simon Cousens, Barrie R. Jones, Adenike Abiose

**Affiliations:** 1 St. John's Institute of Dermatology, London, United Kingdom; 2 Department of Dermatology, Watford General Hospital, West Herts Hospitals NHS Trust, Watford, Herts., United Kingdom; 3 Department of Ophthalmology, Ahmadu Bello University Hospital, Kaduna, Nigeria; 4 International Centre for Eye Health, Institute of Ophthalmology, London, United Kingdom; 5 Department of Clinical Research, Faculty of Infectious and Tropical Diseases, London School of Hygiene and Tropical Medicine, London, United Kingdom; 6 Department of Medicine, Ahmadu Bello University Teaching Hospital, Kaduna, Nigeria; 7 Faculty of Epidemiology and Population Health, London School of Hygiene & Tropical Medicine, London, United Kingdom; 8 National Eye Centre, Kaduna, Nigeria; University of Liverpool, UNITED KINGDOM

## Abstract

**Background:**

*Onchocerca volvulus* infection can result in blindness, itching and skin lesions. Previous research concentrated on blindness.

**Methods:**

A clinical classification system of the cutaneous changes in onchocerciasis was used for the first time in this study within the context of an early ivermectin drug trial in the savanna region of Kaduna State, northern Nigeria. Skin examinations were performed in 6,790 individuals aged 5+ years in endemic communities and 1,343 individuals in nonendemic communities.

**Results / Discussion:**

There was increased risk for all forms of onchocercal skin disease in endemic communities with the most common finding being the presence of nodules (1,438 individuals, 21.2%), followed by atrophy (367, 6.1% of those < 50 years), acute papular onchodermatitis, APOD (233, 3.4%), depigmentation (216, 3.2%) and chronic papular onchodermatitis, CPOD (155, 2.3%). A further 645 individuals (9.5%) complained of pruritus but had completely normal skin. APOD was more common in males whereas atrophy, hanging groin and nodules were more common in females. After controlling for age and sex, microfilarial positivity was a risk factor for CPOD, depigmentation, hanging groin and nodules (OR 1.54, p = 0.046; OR 2.29, p = 0.002; OR 2.18, p = 0.002 and OR 3.80, p <0.001 respectively). Comparable results were found using presence of nodules as the marker for infection. Microfilarial load showed similar, though weaker, results. A total of 2621(38.6%) endemic residents had itching with normal skin, or had one or more types of onchocercal skin disease including nodules, which may be considered as a composite index of the overall prevalence of onchocercal skin disease.

**Conclusion:**

Significant levels of onchocercal skin disease were documented in this savanna area, which subsequently resulted in a reassessment of the true burden of skin disease in onchocerciasis. This paper represents the first detailed report of the association of onchocercal skin disease with markers for onchocercal infection.

## Author summary

Onchocerciasis is a tropical parasitic infection caused by the nematode worm *Onchocerca volvulus*. The disease mainly occurs across tropical Africa and infection can result in blindness, debilitating itching and a variety of skin changes. Initial research concentrated mainly on the problem of blindness. A number of studies on onchocercal skin disease were performed but were difficult to interpret and compare because of the use of inconsistent terminology. Within the setting of one of the early trials of ivermectin in a savanna area of northern Nigeria, where there were known high rates of onchocercal blindness, we used a novel clinical classification of the skin changes in onchocerciasis. We identified significant levels of itching and various forms of onchocercal skin disease within these endemic communities. A positive skin-snip result proved to be a significant risk factor for the presence of chronic papular onchodermatitis (CPOD), depigmentation, hanging groin and onchocercal nodules. Comparable results were found when the presence of nodules was used as the marker for infection and similar, though weaker odds ratios were found with microfilarial load *per se*. The findings triggered a reassessment of the true burden of skin disease in onchocerciasis. It is the first detailed report of the association between onchocercal skin disease and markers of infection.

## Introduction

Onchocerciasis affects approximately 17 million people worldwide [1] with the main burden of disease occurring throughout tropical Africa. The consequences of infection with the nematode *Onchocerca volvulus* include blindness, debilitating pruritus and skin lesions. Initial research concentrated on blindness because of its devastating socio-economic impact. Prior to any control activities in West Africa, it was common to see entire villages near rivers, which were the breeding sites for the *Simulium* vector, completely abandoned for less fertile land elsewhere [2].

It is established that the two epidemiological patterns of ocular onchocerciasis result from two strains of the *O*. *volvulus* parasite. These can be differentiated by DNA sequencing [3;4]. A severe form of ocular disease occurs primarily in savanna areas, where communities often suffer from a high prevalence of onchocercal blindness. Conversely, in rainforest areas onchocercal blindness is less common. The Onchocerciasis Control Programme, OCP (1974–2002) was a large-scale, multi-country programme which aimed to control the vector in countries with high rates of blinding onchocerciasis by regular aerial larviciding of rivers. At its peak, the programme included eleven countries in West Africa and, although expensive, was very successful in interrupting transmission [5].

In 1987, Merck & Co., Inc. announced the donation of Mectizan (ivermectin) for the treatment of onchocerciasis worldwide for as long as necessary. The early large-scale ivermectin trials, including the study described here, were conducted in non-OCP endemic areas, known to have high rates of onchocercal blindness. As ivermectin is mainly microfilaricidal it is necessary to continue annual treatment of the human host population throughout the adult female worms' life-span of up to 14–16 years.

Up until this time, there had been a number of studies on onchocercal skin disease [6–14] but they were difficult to compare because of the lack of a scheme to describe the cutaneous changes. The true burden of skin disease caused by onchocerciasis in any endemic area and globally was therefore unclear. Based on previous field work in Sudan (R.J. Hay, C.D. Mackenzie and J.W. Williams unpublished observations) and in Ecuador [15], a new skin classification scheme [16] was used for the first time in the study described here.

The categories in the clinical classification used for the first time in this study were "consistent with" rather than specific for onchocerciasis. Each subtype has its own clinical differential diagnosis. The objectives of this study were to field test the skin classification scheme on a large scale and to determine the prevalence of onchocerciasis-related skin changes in this savanna setting, a region which was already known to have high rates of onchocercal blindness. In addition, we aimed to explore the association of factors such as age, gender and markers of infection with various forms of onchocercal skin disease.

The data were collected during 1988–1989 prior to commencement of ivermectin therapy. The urgent focus of the work at the time concerned the ocular findings and impact of ivermectin therapy. At the time the skin classification system was published and was adopted for a WHO multi-country study that established the true public health importance of onchocercal skin disease. Following the multi-country study, the African Programme for Onchocerciasis Control, APOC, using community-directed treatment with ivermectin, was established in many countries. Issues such as ivermectin treatment needing to be repeated annually for many years, possible ivermectin resistance and side-effects in areas co-endemic with *Loa-loa* have encouraged the continued search for novel therapies for onchocerciasis. The subsequent widespread use of ivermectin has now rendered our baseline dataset unique in having skinsnip results combined with detailed and validated records of skin pathology in an untreated population. The association of onchocercal skin changes with levels of infection is critical in improving understanding of pathogenesis and aiding the search for new treatments for onchocerciasis. We have therefore further examined the data and report the findings here and wish to ensure the dataset is available to the wider research community.

## Methods

### Ethics statement

The study was approved by the Medical Ethical Committee of Ahmadu Bello University, Zaria, Nigeria, Project Number ESC/89/00024. Approval for the parent project was also approved by the Ministry of Public Health, Nigeria.

### Selection of communities and registration phase

As previously described [17], the study was conducted in the guinea savanna of Kaduna State, northern Nigeria in a subsistence farming mesoendemic area. The initial selection of communities was based on entomological data on *Simulium* blackfly breeding sites, the vectors being *S*. *damnosum ss* [dominant] and *S*. *sirbanum*. In 1988, 36 villages were mapped and residential compounds numbered. All individuals aged 5 years and above were registered and photographed and skin snips taken. The criterion for inclusion of communities in the trial was a microfilarial prevalence of at least 30% among those aged 20 years or above. Thirty four of the communities met the criteria for inclusion in the study. The overall prevalence of positive skin snips among villagers aged 5 years and above was 49% and 72% among those aged 20 years and above.

A further two nonendemic communities with similar ethnic and socio-economic characteristics as the endemic communities were selected in Fatika, Zaria Local Government Area of Kaduna State. These communities served as control communities on the basis of a very low prevalence of onchocercal infection (0.3% positive skin snips in those aged 5 years and above).

### Skin snipping

Two skin snips were taken from both iliac crests in each individual, using a 2mm Holth corneoscleral punch. Snips were incubated for 24–30 hours in normal saline, then weighed and fixed in formal saline. Emergent microfilariae were later counted in Kaduna by independent counters (3 for endemic and 2 for nonendemic communities). In case of disagreement between counters the well was re-examined by an independent observer. The microfilarial load per mg of skin (mf/mg) was calculated for each individual and the community microfilarial load (CMFL) was calculated for each village.

### Skin examinations

Within one year of registration, the study team performed ophthalmic [17] and dermatological examinations on individuals aged 5 years and above. The skin examinations were conducted from 1988–1989. Individuals were asked in the local language (Hausa) about the presence of itching. Skin examinations were conducted privately in natural daylight. The presence or absence of palpable onchocercal nodules and the various types of onchocercal skin disease (OSD) were noted on a form using a standard clinical classification system [16]. Acute papular onchodermatitis (APOD), chronic papular onchodermatitis (CPOD), lichenified papular onchodermatitis (LOD), atrophy, depigmentation (DPM), hanging groin and onchocercal nodules were documented. APOD, CPOD and LOD were collectively termed reactive skin lesions. In order to avoid confusion with senile or age-related atrophy of the skin, onchocercal skin atrophy was only recorded as an abnormal finding in individuals aged less than 50 years. The categories in the clinical classification are "consistent with" rather than specific for onchocerciasis and each subtype has its own clinical differential diagnosis [16]. The presence of non-onchocercal skin disease was also recorded. All skin examinations were conducted masked to skin snip and eye examination results.

### Consent

Following preliminary discussions with the village heads, informed consent for skin snipping and subsequent eye and skin examinations was obtained in the local Hausa language and confirmed with a signature or finger-print.

### Data analysis

Data were double-entered onto computers with the software package DBase III+ and were cleaned with DBase III+ and SAS/PC. Analyses were done using STATA/IC 12.0 (http://www.stata.com). Logistic regression, both univariable and multivariable, was undertaken to investigate the association between various forms of onchocercal skin disease and onchocercal infection (using microfilarial (mf) positivity, microfilarial load and nodules as separate markers), age and gender. Account was taken for clustering by village in all analyses by using linearization-based variance estimators.

## Results

### Inter-observer variation study

A substantial effort was made to minimise inter-observer variation before the start of data collection. An inter-observer variation study for recording itching and each of the categories of onchocercal skin disease was conducted under similar lighting conditions by two general physicians (HNY and NN) on 291 individuals. Good Kappa values were obtained as follows: pruritus with clinically normal skin = 0.68 (95% confidence intervals (CI) 0.59–0.76); APOD = 0.72 (CI 0.60–0.85); CPOD = 0.79 (CI 0.56–1.03); LOD = 1.0 (CI 0.98–1.02); atrophy = 0.84 (0.68–1.0) and nodules = 0.83 (0.75–0.90). There were insufficient numbers of individuals with DPM (n = 3) or hanging groin (n = 3) to allow comparision. However, these are two of the most easily identified forms of onchocerciasis-related pathology.

### Endemic onchocercal communitites

A total of 8,140 individuals aged 5 years and above were registered at the time of the census and 7,072 were present at the time of examinations one year later. Of these 6,790 consented to a skin examination. Skin snip data was available for 6,643 (97.8% of those examined, Table 1). Overall 3,276 (49.3% of those snipped) had positive skin snips. Of those who were skin snip positive, the majority (1872/3276, 57.1%) had low microfilarial loads (≤10.00 mf/mg skin).

**Table 1:** Characteristics of residents who underwent skin examinations in endemic and nonendemic villages showing age, sex and microfilarial load (mf/mg)*.

	Endemic villages N = 6790				Nonendemic villages N = 1343			
	Male		Female		Male		Female	
Age (years)	N	mf/mg	N	mf/mg	N	mf/mg	N	mf/mg
5–14	1334	1.5	1189	0.5	266	0	233	0
15–24	560	12.7	679	6.7	135	0	164	0
25–34	478	14.0	622	10.8	95	0	118	0
35–44	409	13.4	439	13.5	78	0.004	71	0
45–54	293	12.9	290	12.5	50	0	44	0
55–64	150	16.3	155	10.4	27	0	24	0
65+	126	13.9	66	13.3	24	0	14	0

* Number (%) skin-snipped: Endemic villages = 6,643 (97.8%); Nonenendemic villages = 1,342 (99.9%)

### Non-endemic communities

A total of 1,886 individuals aged 5 years and above were registered at the time of census. Skin examinations were conducted on 1,343 individuals. Skin snip data was available for 1,342 (99.9% of those examined, Table 1). Only 4 persons (0.3%) in the non-endemic villages had positive skin snips, all with microfilarial loads ≤10.00 mf/mg skin.

### Onchocerciasis-related skin changes

The prevalences of skin changes consistent with onchocerciasis in endemic and nonendemic communities are summarised in Table 2. In endemic communities the most common clinical sign was the presence of palpable onchocercal nodules in 1438 (21.2%) of the examined population. The next most frequent finding was atrophy (367; 6.1% of those aged <50 years) followed by APOD (233, 3.4%), depigmentation (216, 3.2%) and CPOD (155, 2.3%). Overall, including nodules, 1976 (29.1%) persons had one or more forms of OSD. A further 645 individuals (9.5%) complained of pruritus but had completely normal skin. A total of 2621(38.6%) of the examined endemic population were found to complain of itching with normal skin or had one or more types of OSD including nodules, which may be considered as a composite index of the overall prevalence of onchocercal skin disease.

**Table 2:** Pre-control prevalence of onchocercal skin disease in endemic and nonendemic villages.

Skin condition	Endemic villages N = 6790		Nonendemic villages N = 1343		Univariable OR^d^ (95% CI)	P value
	n	% (95% CI)	n	% (95% CI)		
APOD*	233	3.4 (2.2–4.7)	5	0.4 (0.0–1.0)	9.509 (6.187–14.615)	<0.001
CPOD*	155	2.3 (1.7–2.9)	11	0.8 (0.0–3.5)	2.829 (1.762–4.542)	<0.001
LOD*	5	0.1 (0.0–0.16)	0	0	-	-
**Reactive skin lesions** (i.e. APOD+/-CPOD+/-LOD*)	351	5.2 (3.7–6.6)	15	1.1 (0.7–2.9)	4.826 (3.399–6.853)	<0.001
**Atrophy** (Individuals aged <50 yrs)^a^	367	6.1 (4.8–7.4)	47	3.9 (0.0–8.3)	1.611 (1.233–2.106)	= 0.001
**Depigmentation**^b^	216	3.2 (2.6–3.8)	4	0.3 (0.00–1.2)	10.999 (7.362–16.433)	<0.001
**Hanging Groin**	95	1.4 (1.0–1.8)	1	0.1 (0.0–0.3)	19.043 (11.680–31.047)	<0.001
**Nodules**	1438	21.2 (18.0–24.4)	4	0.3 (0.0–1.3)	89.942 (58.731–137.738)	<0.001
**Any of the above**	1976	29.1 (25.8–32.4)	68	5.1 (0.0–10.5)	7.696 (6.271–9.445)	<0.001
**Itching with clinically normal skin**^c^	645	9.5 (5.5–13.5)	25	1.9 (0.0–8.0)	5.534 (3.029–10.109)	<0.001
**Any of the above**	2,621	38.6 (32.7–44.5)	93	6.9 (0.0–18.5)	8.450 (6.117–11.672)	<0.001

* APOD = Acute Papular Onchodermatitis; CPOD = Chronic Papular Onchodermatitis; LOD = Lichenified Onchodermatitis

^**a**^ Denominator for individuals aged <50 yrs in endemic villages = 6,022; nonendemic villages = 1,214

^b ^Depigmentation includes pale brown leopard skin plus complete depigmentation or typical leopard skin

^c ^No evidence of onchocercal skin disease, nor any other itchy skin disease

^d ^Account was taken for clustering by village in all analyses. Multivariable OR, corrected for age group and sex revealed similar results (S1 Table).

In the nonendemic communities all forms of onchocercal skin disease and the presence of itching with normal skin were rare. No cases of LOD were identified. The most common finding potentially associated with onchocercal infection was atrophy in 47 persons (3.9% of those aged < 50 years).

There was an increased risk for all forms of OSD in endemic compared with nonendemic communities. The highest risk was seen for the presence of nodules (OR 89.94), followed by hanging groin (OR 19.04), depigmentation (OR 11.0), APOD (OR 9.51), Reactive Skin Lesions (OR 4.83), CPOD (OR 2.83, all p<0.001) and atrophy (OR 1.6, p = 0.001). Endemic communities also had a higher risk of itching alone with clinically normal skin (OR 5.53) and itching alone or one or more OSD- associated findings including nodules (OR 8.45, both p<0.001).

### Age and gender-specific prevalence of OSD-associated findings in endemic communities

**Fig 1:**
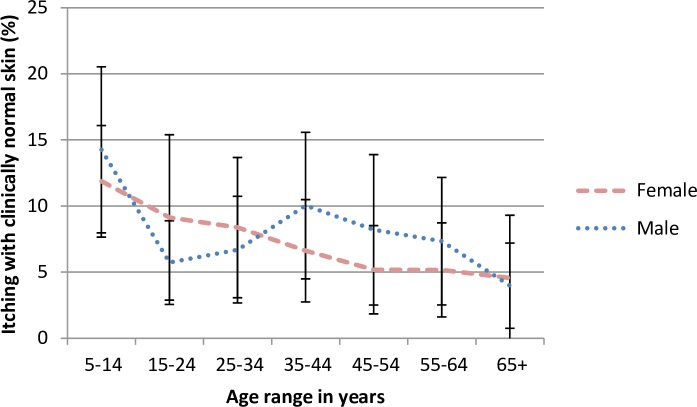
Age and gender-specific prevalence of itching with clinically normal skin in endemic savanna communities, Kaduna State, Northern Nigeria. Error bars represent mean and 95% CI.

**Fig 2:**
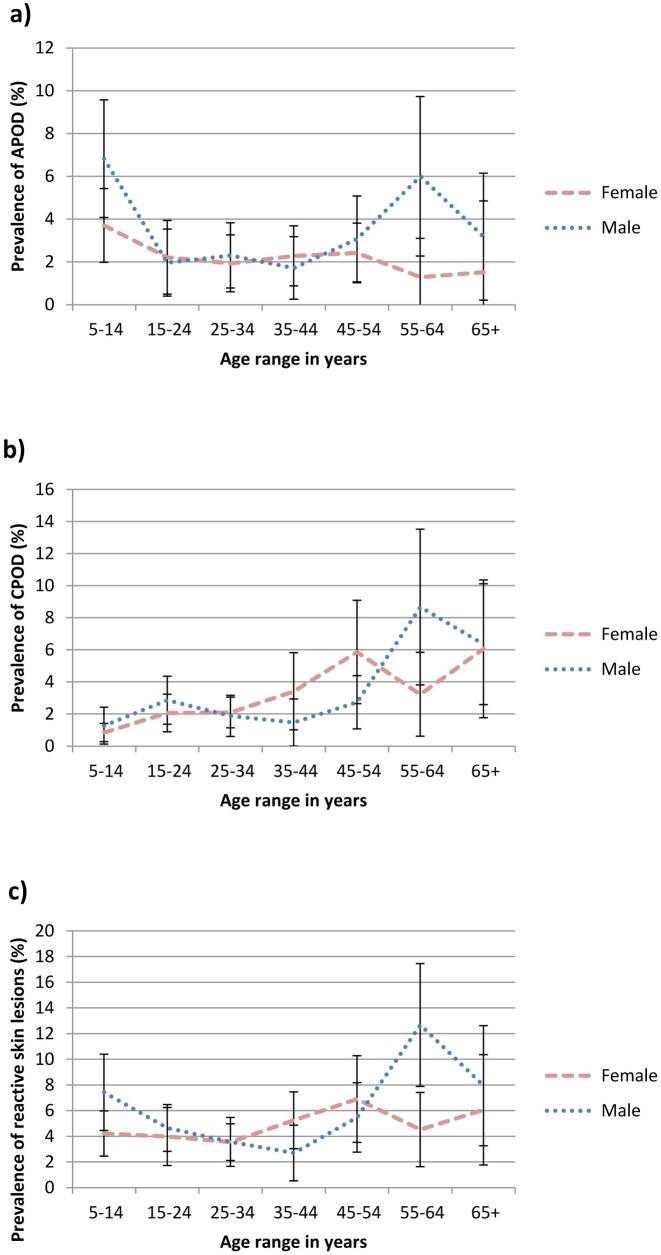
Age and gender-specific prevalence of a) acute papular onchodermatitis, b) chronic papular onchodermatiits and c) reactive skin lesions (i.e. acute papular onchodermatitis +/- chronic papular onchodermatitis +/- lichenified onchodermatitis) in endemic savanna communities, Kaduna State, Northern Nigeria. Error bars represent mean and 95% CI.

**Fig 3:**
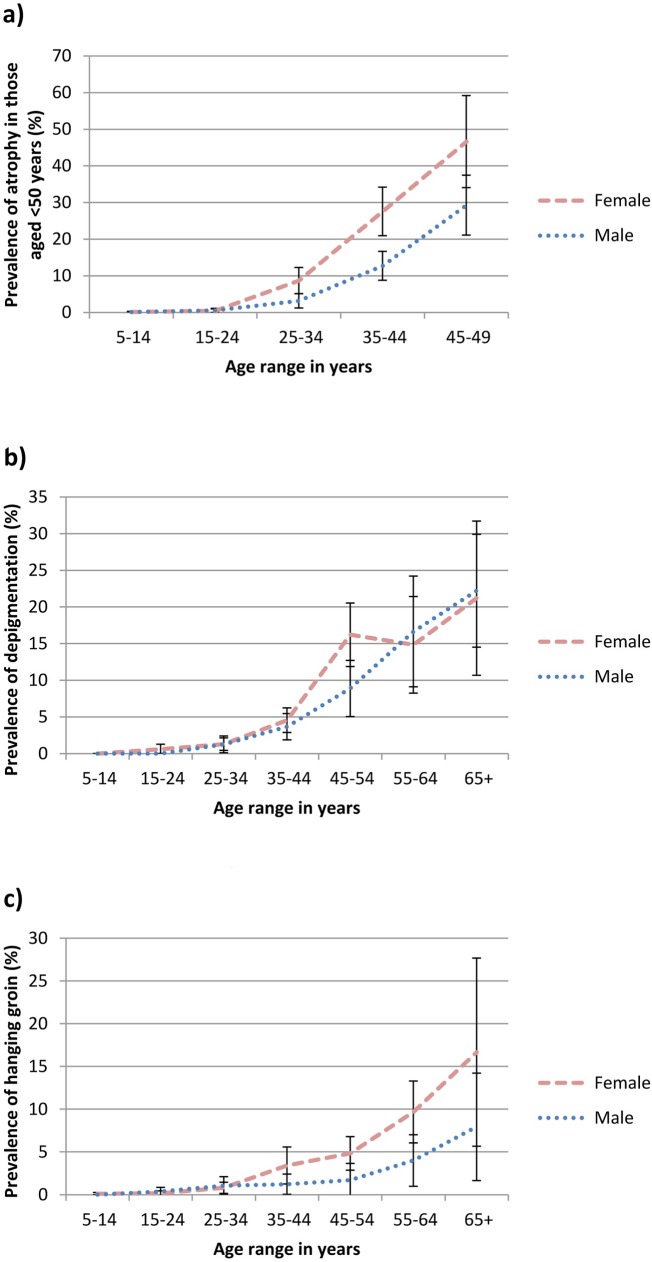
Age and gender-specific prevalence of a) atrophy in individuals aged < 50 years, b) depigmentation and c) hanging groin in endemic savanna communities, Kaduna State, Northern Nigeria. Error bars represent mean and 95% CI.

**Fig 4:**
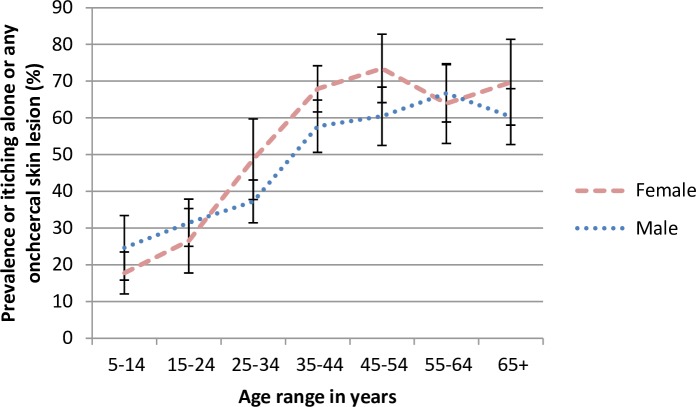
Age and gender-specific prevalence of itching alone or any onchocercal skin disease including nodules in endemic savanna communities, Kaduna State, Northern Nigeria. Error bars represent mean and 95% CI.

**Fig 5:**
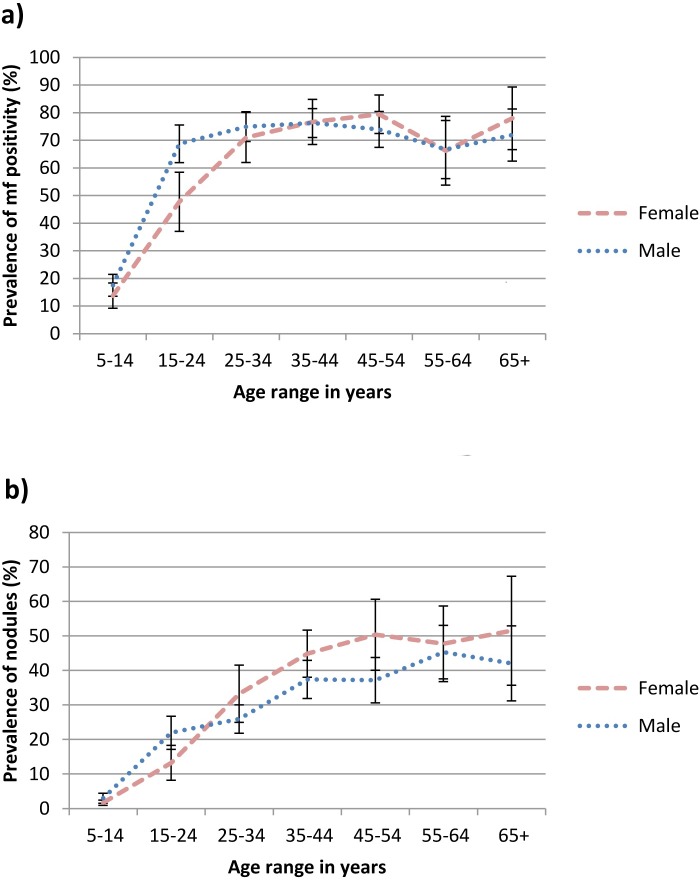
Age and gender-specific prevalence of a) microfilarial positivity and b) nodules in endemic savanna communities, Kaduna State, Northern Nigeria. Error bars represent mean and 95% CI.

Figs 1–5 show age and gender-specific prevalence of onchocercal skin disease and markers of onchocercal infection in endemic communities. Fig 1 shows the age and gender-specific prevalence for itching with clinically normal skin. The highest prevalence was seen in the youngest age group of 5–14 years, with an overall trend to reduce with age.

The age and gender-specific prevalence of APOD, CPOD and reactive onchocercal skin lesions in endemic communities are shown in Fig 2. There were only five cases of LOD, four of whom were female. Three were aged 15–24 and the remaining two were older. The highest prevalence of APOD was seen in those aged 5–14. Whilst the prevalence was fairly constant in older females, it possibly rose again in males aged 45 years or more. CPOD was more common in those aged 45 years or more. The overall prevalence of reactive skin disease reflects these findings. Among females there was a small rise in prevalence with age. Among males prevalence was lowest between 15 and 44 years of age.

Fig 3 shows the age and gender distribution of markers of chronic disease. All markers of chronic disease increased strongly with age: atrophy and depigmentation from 25 years onwards and hanging groin from 35 years onwards. Atrophy and hanging groin had a consistently higher prevalence in females.There was little gender difference with depigmentation.

A composite index of the overall prevalence of onchocercal skin disease was created of itching alone or any onchocercal skin lesion including nodules. Fig 4 shows the age and gender-specific prevalence of this index. There was a marked increase in prevalence with age for both sexes, with a trend for prevalence in females aged 25 years and above. In both sexes the prevalence plateaued from age 35 years onwards, reaching around 70% in females and 60% in males.

Fig 5 shows age- and gender-specific prevalence of infection as determined by mf positivity and presence of nodules. The prevalence of mf positivity rose steeply with age, reaching a plateau of approximately 70% from the age of 15 years onwards in males and 25 years onwards in females. In contrast there was a steady rise in nodule prevalence until the age of 45 years. In the older age groups nodules were consistently more prevalent in females. Nodule prevalence plateaued at approximately 40% in males and 50% in females.

The prevalence of the various types of onchocercal skin disease were examined by two markers of onchocercal infection, skin snip positivity and presence of palpable onchocercal nodule (S2 Table). CPOD, atrophy, depigmentation and hanging groin were more prevalent among those with positive skin snips and among those with nodules. Neither APOD nor itching without skin lesions were more common among those with these markers of onchocercal infection.

### Logistic regression analyses

Multivariable logistic regression analyses were performed to investigate the association of each form of onchocercal skin disease with infection after controlling for age and gender (Table 3). Three models were created using mf positivity, mf load or nodules as the marker of infection. Mf positivity was associated with increased risk of CPOD (OR = 1.54, p = 0.046), depigmentation (OR = 2.29, p = 0.002), hanging groin (OR = 2.18, p value = 0.002) and nodules (OR = 3.80, p<0.001).

**Table 3:** Univariable and multivariable logistic regression analyses for itching alone and onchocercal skin disease outcomes in endemic villages. Account was taken for clustering by village in all analyses. Each of the multivariate models included risk factors of age^a^, sex^b^, plus either *i)* mf positivity, *ii)* mf load or *iii)* presence of nodules as the marker for infection.

Outcome	Risk Factor	Univariable OR (95% C I)	p	Multivariable OR (95% C I) using mf positivity	p
**Itching alone**	**Mf positivity**	0.638 (0.414–0.985)	= 0.043	0.843 (0.591–1.202)	= 0.335
	**Mf load**^**c**^	0.705 (0.551–0.901)	= 0.007	0.802 (0.661–0.973)	= 0.026
**APOD**	**Mf positivity**	0.705 (0.494–1.005)	= 0.053	1.121 (0.725–1.734)	= 0.597
	**Mf load**	0.798 (0.655–0.973)	= 0.027	1.014 (0.807–1.272)	= 0.905
	**Nodules**^**d**^	0.718 (0.477–1.081)	= 0.109	1.097 (0.679–1.773)	= 0.697
**CPOD**	**Mf positivity**	2.267 (1.475–3.484)	<0.001	1.536 (1.008–2.341)	= 0.046
	**Mf load**	1.402 (1.182–1.662)	<0.001	1.160 (0.947–1.422)	= 0.146
	**Nodules**	2.342 (1.781–3.081)	<0.001	1.589 (1.097–2.300)	= 0.016
**Reactive Skin Lesions**	**Mf positivity**	1.048 (0.757–1.452)	= 0.769	1.209 (0.848–1.724)	= 0.283
	**Mf load**	1.008 (0.850–1.195)	= 0.923	1.066 (0.880–1.291)	= 0.504
	**Nodules**	1.221 (0.912–1.634)	= 0.173	1.351 (0.951–1.919)	= 0.091
**Atrophy (<50 yrs)**	**Mf positivity**	4.993 (3.690–6.755)	<0.001	1.390 (0.941–2.052)	= 0.095
	**Mf load**	1.940 (1.741–2.162)	<0.001	1.241 (1.047–1.470)	= 0.014
	**Nodules**	4.597 (3.777–5.595)	<0.001	1.534 (1.206–1.950)	= 0.001
**DPM**	**Mf positivity**	6.183 (3.873–9.871)	<0.001	2.289 (1.377–3.804)	= 0.002
	**Mf load**	1.990 (1.767–2.242)	<0.001	1.426 (1.229–1.655)	<0.001
	**Nodules**	4.793 (3.699–6.209)	<0.001	1.699 (1.251–2.308)	= 0.001
**Hanging groin**	**Mf positivity**	5.594 (3.595–8.703)	<0.001	2.179 (1.375–3.454)	= 0.002
	**Mf load**	1.823 (1.674–1.985)	<0.001	1.297 (1.138–1.478)	<0.001
	**Nodules**	7.263 (4.532–11.64)	<0.001	2.760 (1.647–4.627)	<0.001
**Nodules**	**Mf positivity**	8.234 (6.031–11.24)	<0.001	3.800 (2.539–5.689)	<0.001
	**Mf load**	2.631 (2.368–2.923)	<0.001	1.858 (1.615–2.137)	<0.001
**Itching alone or any OSD**	**Mf positivity**	3.460 (2.607–4.593)	<0.001	2.209 (1.715–2.844)	<0.001
	**Mf load**	1.974 (1.722–2.263)	<0.001	1.564 (1.385–1.766)	<0.001

^a^Age groups = 5–14,15–24,25–34,35–44,45–54,55–64 and 65+ years

^b^Sex index = female

^c^mf load = 0,0.01–10.00,10.01–50.00,50.01–100.00 and >100mf / mg skin

^d^Nodules = present or absent

Comparable increased risks were noted when the presence of nodules was used as the marker of infection (CPOD OR = 1.59, p = 0.016; depigmentation OR = 1.70, p = 0.001; hanging groin OR = 2.76, p <0.001). Nodules were also a risk factor for atrophy (OR 1.53, p = 0.001).

Similar, though weaker, increased risks were found when mf load *per se* was used as the marker of infection (atrophy OR = 1.24, p = 0.014; depigmentation OR = 1.43, p <0.001; hanging groin OR = 1.30, p <0.001; and nodules OR = 1.86, p <0.001).

After controlling for age and sex in the multivariable analysis, mf load was protective for the presence of itching alone with clinically normal skin (OR 0.80, p = 0.026). After controlling for age and sex there were no significant associations for APOD with microfilarial positivity, mf load or presence of nodules.

In the multivariate analysis the composite index of onchocercal skin disease (itching alone with clinically normal skin or any onchocercal skin disease including nodules) was significantly associated with mf positivity (OR = 2.21, p<0.001). The association was weaker if mf load was used as the marker of infection (OR = 1.56, p<0.001).

### Prevalence of non-onchocercal skin disease

The prevalence of non-onchocercal skin disease by meso- and nonendemic communities is shown in Table 4. Overall the prevalence of non-onchocercal skin disease was lower in the endemic communities (53.7% vs 69.1%, OR 0.52, p = 0.024).

**Table 4:** Prevalences of non-onchocercal skin diseases in endemic and nonendemic villages.

Skin condition	Endemic villages N = 6790		Nonendemic villages N = 1343		Univariable OR^a^ (95% CI)	P value
	n	% (95% CI)	n	% (95% CI)		
**Acne**	970	14.3 (9.4–19.2)	381	28.4 (5.0–51.7)	0.421 (0.276–0.642)	<0.001
**Pyoderma**	622	9.2 (7.3–11.0)	44	3.3 (2.8–3.8)	2.977 (2.373–3.736)	<0.001
**Scabies**	298	4.4 (1.4–7.4)	14	1.0 (0.0–4.5)	4.357 (1.950–9.739)	= 0.001
**Pityriasis versicolor**	234	3.4 (2.4–4.5)	78	5.8 (0.0–20.6)	0.579 (0.372–0.900)	= 0.017
**Miliaria**	203	3.0 (0.0–4.6)	7	0.5 (0.0–6.4)	5.882 (1.441–24.015)	= 0.015
**Dermatophyte infection**	96	1.4 (0.8–2.1)	33	2.5 (1.1–3.8)	0.569 (0.358–0.905)	= 0.019
**Insect bites**	29	0.4 (0.3–0.6)	16	1.2 (0.0–2.9)	0.356 (0.233–0.543)	<0.001
**Other skin diseases**	2,350	34.6 (24.5–44.7)	669	49.8 (14.2–85.4)	0.533 (0.332–0.857)	= 0.011
**Any non-OSD**	3,644	53.7 (40.4–67.0)	928	69.1 (33.0–100)	0.518 (0.294–0.913)	= 0.024

^a ^Account was taken for clustering by village in all analyses. Multivariable OR, corrected for age group and sex revealed similar results (S3 Table).

The most common non-onchocercal skin disease was acne which was only half as prevalent in endemic communities. In contrast pyoderma, scabies and miliaria were more prevalent in the endemic communities.

Findings of pityriasis versicolor, dermatophyte infection and insect bites were all less common in the endemic communities. A large number of other skin conditions were also recorded, which were generally less prevalent in the endemic communities. These included keloids, eczema, burn scars, warts and erythema ab igne.

## Discussion

This study reports the first use of the classification of the cutaneous changes associated with onchocerciasis on a large scale. It is worth emphasizing that this scheme, which is based purely on clinical signs, was applied without the physician being aware of the individual's skin snip status or results of their eye examination. The clinical signs of each type of onchocercal skin disease are relatively non-specific and hence each category has its own list of clinical differential diagnoses [16]. The most commonly observed onchocercal skin finding in the endemic communities was onchocercal nodules (21.2%) followed by cutaneous atrophy (6.1% of individuals aged <50 years), APOD (3.4%) and depigmentation (3.2%). LOD was rare in this savanna area with only five cases identified. Careful clinical examination highlighted a further group of individuals (9.5% of endemic residents) who complained of itching but had clinically normal skin. Thus, within this endemic region, a total of 38.6% of the population aged 5 years and above had itching with normal skin or one or more forms of OSD including nodules. This demonstrates a remarkably high overall prevalence of onchocercal skin disease in these communities. Although savanna areas of sub-Saharan Africa were known to have high burdens of blinding onchocercal eye disease, this was the first time high levels of onchocercal skin disease had been documented in a savanna region.

The prevalence of onchocercal skin disease in these communities is an underestimate since a diagnosis of atrophy was limited to those aged less than 50 years in order to avoid confusion with senile atrophy of the skin. APOD, CPOD and LOD are by definition, itchy conditions and the degree of the burden of itching suffered by these residents has not been captured. The true prevalence of onchocercal-induced atrophy and itching in the community will therefore be higher.

As mentioned previously, the various skin changes consistent with onchocerciasis may be clinically non-specific. The strong positive associations of CPOD, depigmentation and hanging groin with microfilarial positivity, independent of age and sex, show these classification sub-groupings are relevant to onchocercal infection. It is possible that the relationship of these clinical findings with onchocercal infection may be even stronger since it is known that skin snip sensitivity is increased with higher numbers of skin snips. Sensitivity for infection may be even further increased by the use of PCR or LAMP of skin snips [18,19]. CPOD, atrophy, depigmentation and hanging groin were also associated with the presence of nodules, and atrophy, depigmentation, hanging groin and nodules were all associated with microfilarial load.

It is possible that individuals with pruritus but clinically normal skin had early, light infections which were not always detectable by the routine number of two skin snips performed in this study and this may explain why no association with mf positivity could be documented in the multivariable regression analyses. Paradoxically mf load was found to be inversely associated with itching alone. The reason for this is unclear.

Similarly APOD, which was more common in 5–14 year olds who presumably also had early, light, onchocercal infections, did not show associations with infection in this study. Microfilarial remnants have previously been demonstrated in epidermal microabscesses in skin biopsies of APOD (Murdoch *et al*. Brit J Dermatol 1990: 123 (Suppl 37):28).

There were only five cases of LOD, three of whom were microfilaria positive. LOD is associated with hyperimmune host immune responses, skin snips are often negative and microfilariae are difficult to find on skin biopsy [11]. Atrophy, hanging groin and nodules were all more common in females.

There were only 4 persons skin snip positive in the nonendemic villages. The prevalence of all forms of skin disease consistent with onchocercal infection was very low in nonendemic villages, supporting the clinical classification scheme as consistent with onchocercal infection. A caveat is that the clinical observers were aware that they were in a nonendemic area. It is possible that this may have produced an element of observer bias, but the observers were masked to all skin snip and eye examination results. The higher prevalence of pyoderma in endemic communities might be a result of pruritus due to either onchocerciasis or the higher prevalence of scabies causing excessive excoriation and secondary bacterial infection. The increased prevalence of other skin diseases in the nonendemic communities might be explained by small changes such as a wart or keloid being easier to see if the skin was otherwise clear.

At all ages, microfilarial positivity was an earlier and more sensitive marker of onchocercal infection than prevalence of nodules (Fig 5). Rapid epidemiological mapping of onchocerciasis (REMO) followed by rapid epidemiological assessment (REA), the examination of samples of 30–50 adult men for the presence of nodules [20,21], is now a well established and useful method to quickly assess levels of onchocercal endemicity in areas to decide priorities for mass drug treatment. Nodule palpation in adult males underestimates the prevalence of infection compared with the more time-consuming and costly process of skin snipping and quantitative models have been developed to describe the association and estimate microfilarial `prevalence from measured nodule prevalence [22]. The results presented here suggest a possible further underestimate as adult females consistently carry the larger burden of disease due to nodules. The practical difficulties, however, of undressing women in privacy for palpation of nodules probably outweigh any benefits that might be gained from changing practice.

Since this study was conducted, the skin classification scheme been successfully used in rain-forest onchocerciasis-endemic areas in Africa [23] as well as in a variety of mass drug treatment [24;25], psychosocial and economic [26–28], genetic [29] and immunological studies [30;31].

The multi-country rainforest study [23], which used prevalence of nodules as a marker of endemicity, confirmed that onchocercal skin disease was a significant public health problem in affected areas with an overall prevalence of onchocercal skin lesions (excluding nodules) in those aged 5 years and above of 28%. The prevalence of APOD, CPOD, LOD and depigmentation was higher in the rainforest areas compared to the findings reported here from a savanna region. Excluding nodules, the most common form of OSD in rainforest areas was CPOD at 13%, whereas atrophy was the most common in this savanna area. Interestingly atrophy was the only type of OSD which was more common in the savanna than rain-forest regions.

This savanna study and the later multicountry rainforest skin survey prompted a reassessment of the skin disease burden of onchocerciasis. The rainforest study's results raised the possibility that many other endemic rainforest areas across Africa had significant levels of onchocercal skin disease, and hence merited mass drug treatment, even though they had low levels of blinding onchocercal eye disease. In 1995, a new control programme, the African Programme for Onchocerciasis Control (APOC), was launched. APOC used a sustainable strategy of community-directed treatment with ivermectin (CDTI) whereby the communities themselves implemented annual ivermectin distribution. APOC covered 20 countries and closed in 2015.

A multicountry skin survey after five or six years of annual ivermectin therapy in meso-and hyperendemic communities [25] revealed significant reductions in the odds ratios of itching (with and without accompanying OSD), APOD, CPOD, LOD, reactive skin lesions, depigmentation and nodules. Atrophy was not assessed.

The aim of the skin classification system is to facilitate standardisation of data collected by different observers in different geographical settings and enable comparisons of results. Observers in the multi-country rainforest study [23] were all trained by the same author (MM) and underwent an inter-observer variation study prior to data collection. Furthermore several of the same clinicians collected the data in the multi-country study performed after five or six years of ivermectin therapy [25], again following an inter-observer variation study. It is reasonable to claim therefore that the results from the current study and from these latter two studies are comparable.

In contrast to the Onchocerciasis Elimination Programme in the Americas (OEPA), which aimed to eliminate onchocerciasis from foci in Central and Southern America [32], APOC's original objective was to try to control onchocerciasis as a public health problem. It was unclear whether ivermectin could interrupt transmission and eventually eradicate onchocerciasis in Africa where the vectors were known to be more efficient. Studies in Mali and Senegal [33], however, have shown that after 15–17 years of annual or six monthly ivermectin therapy the prevalence of microfilariae and vector infectivity rates were either zero or below postulated thresholds for elimination, which triggered revision of APOC's stated objective to one of elimination of onchocerciasis in Africa. It is exciting to note that follow up studies in the same savanna communities reported here reveal that after 15–17 years of annual ivermectin therapy the community prevalence of mf positivity has fallen to 0%. All 3,703 individuals examined were skin snip negative [34]. This represents the first evidence from an APOC operational area that ivermectin treatment alone could eliminate onchocerciasis infection and potentially disease transmission in endemic areas in Africa.

The need for repeated treatments of ivermectin over many years has led to concerns of development of ivermectin resistance [35]. Research continues for a macrofilaricidal drug [36,37].

The pathogenesis of the cutaneous changes in onchocerciasis is still not fully understood. There is a spectrum of immune response to infection, with some infected persons showing a minimal immune response to parasite antigens, allowing the proliferation of microfilariae in the absence of clinical symptoms, while others have an intact and symptomatic immune response [38]. An immunogenetic basis for this clinical spectrum has been suggested [29;39;40] and differing isotypic antibody responses [30] and cellular immune responses [31;41–45] may play a role. The endosymbiotic bacteria *Wolbachia* are essential for the pathogenesis of *O*. *volvulus* keratitis in a mouse model [46]. The relative *Wolbachia* DNA burden was previously thought to explain the difference in ocular pathogenicity of the two strains of *O*. *volvulus* [47] but recent whole-genome data challenges this concept [48]. It is hoped that improved understanding of the pathogenesis of onchocercal skin disease, including clinico-pathological correlations of how it is related to human host age, sex and microfilarial load as delineated here, may help in the endeavour to identify novel treatments.

The Global Burden of Disease Study has estimated 15,531,500 prevalent cases of onchocerciasis remaining in 2015, representing a 29.1% reduction in global prevalence since 2005. Based on the skin clinical classification, the disease burden from onchocercal skin disease has been now included alongside onchocercal eye disease to form an overall global estimate of 1,135,700 years lived with disability (YLDs) due to onchocercal infection [49].

In summary the skin classification scheme for the cutaneous changes in onchocerciasis was easy to use in the field, reproducible and a useful tool to assess the prevalence of onchocerciasis skin disease in this savanna region of northern Nigera. We report that the most common onchocercal skin finding was nodules, followed by atrophy, APOD, depigmentation and CPOD. APOD was more common in males whereas atrophy, hanging groin and nodules were more common in females. Microfilarial positivity and the presence of nodules were associated with CPOD, depigmentation and hanging groin. Nodules were also a risk factor for atrophy whereas microfilarial load showed similar, though weaker associations.

The use of the skin classification scheme in other prevalence and socio-economic studies has contributed towards an ever-growing body of research which aims to estimate the true global disease burden of onchocerciasis, which takes into account not only ophthalmological, but also cutaneous effects of the disease on its unfortunate sufferers.

## Supporting information

S1 File Data set for study. (DTA)

S2 File Code booklet for data set. (DOCX)

S3 File STARD checklist. (DOCX)

S1 Table Pre-control prevalence of onchocercal skin disease in endemic and nonendemic villages (multivariate analysis correcting for age and gender). (DOCX)

S2 Table Prevalence of onchcercal skin disease related to indicators of onchocercal infection (DOCX)

S3 Table Prevalences of non-onchcercal skin disease in endemic and nonendemic villages (multivariate analysis correcting for age and gender). (DOCX)
