# Supplementary material for: Pre-control relationship of onchocercal skin disease with onchocercal infection in Guinea Savanna, Northern Nigeria
Source: PLoS Negl Trop Dis. 2017 Mar 29;11(3):e0005489. doi: 10.1371/journal.pntd.0005489 (PMC5386293; doi:10.1371/journal.pntd.0005489)
Supplement: S2 File — (DOCX) [file pntd.0005489.s002.docx]

**Code Book for Dataset**

Age = age in years at time of examination (numeric)

Sex = gender

0=female

1= male

Vcode = village code (numeric)

Mfbin = microfilaria count in skin snip

0 = nil

1 = >0<10

2 = 10>50

3 = 50>100

4 = 100+

mesonon = community type

0 = nonendemic community

1 = mesoendemic community

All fields below have code = 0 for absence of the sign/symptom and code = 1 for presence of the sign/symptom.

Atrophy = presence of skin atrophy in those aged under 50 years of age

Nodules = presence of palpable onchocercal nodules

Itching = presence of troublesome/symptomatic itching

APOD = presence of signs compatible with acute papular onchodermatitis

CPOD = presence of signs compatible with chronic papular onchodermatitis

LOD = presence of signs compatible with lichenified onchodermatitis

Hypo = presence of signs compatible with onchocercal depigmentation

Hanggroin = presence of signs compatible with hanging groin

Anyotherskin = presence of signs of non-onchocercal skin pathology including acne, pyoderma, scabies, pityriasis versicolor, miliaria, dermatophyte infection, insect bites and other non-onchocercal skin diseases
