# Supplementary material for: Pre-control relationship of onchocercal skin disease with onchocercal infection in Guinea Savanna, Northern Nigeria
Source: PLoS Negl Trop Dis. 2017 Mar 29;11(3):e0005489. doi: 10.1371/journal.pntd.0005489 (PMC5386293; doi:10.1371/journal.pntd.0005489)
Supplement: S1 Table — (DOCX) [file pntd.0005489.s004.docx]

**S1 Table Pre-control prevalence of onchocercal skin disease in endemic and nonendemic villages (multivariate analysis correcting for age and gender).**

| **Skin condition** | **Endemic villages N=6790** | | | **Nonendemic villages N=1343** | | **MultivariableOR^d^ (95% CI)** | **P value** |
| --- | --- | --- | --- | --- | --- | --- | --- |
|  | **n** |  | **% (95% CI)** | **n** | **% (95% CI)** |  |  |
| **APOD*** | 233 |  | 3.4 (2.2-4.7) | 5 | 0.4 (0.0-1.0) | 9.574 (6.203-14.778) | <0.001 |
| **CPOD*** | 155 |  | 2.3 (1.7-2.9) | 11 | 0.8 (0.0-3.5) | 2.772 (1.715-4.482) | <0.001 |
| **LOD*** | 5 |  | 0.1 (0.0-0.16) | 0 | 0 | - | - |
| **Reactive skin lesions** (i.e. APOD+/-CPOD+/-LOD*) | 351 |  | 5.2 (3.7-6.6) | 15 | 1.1 (0.7-2.9) | 4.803 (3.361-6.862) | <0.001 |
| **Atrophy** (Individuals aged <50 yrs)^a^ | 367 |  | 6.1 (4.8-7.4) | 47 | 3.9 (0.0-8.3) | 1.487 (1.072-2.063) | =0.001 |
| **Depigmentation**^b^ | 216 |  | 3.2 (2.6-3.8) | 4 | 0.3 (0.00-1.2) | 10.777 (7.089-16.384) | <0.001 |
| **Hanging Groin** | 95 |  | 1.4 (1.0-1.8) | 1 | 0.1 (0.0-0.3) | 17.931 (10.932-29.411) | <0.001 |
| **Nodules** | 1438 |  | 21.2 (18.0-24.4) | 4 | 0.3 (0.0-1.3) | 104.243 (66.376-163.711) | <0.001 |
| **Any of the above** | 1976 |  | 29.1 (25.8-32.4) | 68 | 5.1 (0.0-10.5) | 9.231 (7.243-11.765) | <0.001 |
| **Itching with clinically normal skin**^c^ | 645 |  | 9.5 (5.5-13.5) | 25 | 1.9 (0.0-8.0) | 5.597 (3.070-10.202) | <0.001 |
| **Any of the above** | 2,621 |  | 38.6 (32.7-44.5) | 93 | 6.9 (0.0-18.5) | 9.751 (6.799-13.983) | <0.001 |
