# Supplementary material for: Pre-control relationship of onchocercal skin disease with onchocercal infection in Guinea Savanna, Northern Nigeria
Source: PLoS Negl Trop Dis. 2017 Mar 29;11(3):e0005489. doi: 10.1371/journal.pntd.0005489 (PMC5386293; doi:10.1371/journal.pntd.0005489)
Supplement: S2 Table — (DOCX) [file pntd.0005489.s005.docx]

**S2 Table Prevalence of onchocercal skin disease related to indicators of onchocercal infection**

|  | **Mesoendemic villages** | | | | | | **Nonendemic villages** | | | | | |
| --- | --- | --- | --- | --- | --- | --- | --- | --- | --- | --- | --- | --- |
|  | **mf-ve**  **n=3367** | **mf+ve (%)**  **n=3276** | **Total**  **N=6643** | **Nodules**  **-ve n=5389** | **Nodules +ve (%) n=1449** | **Total**  **N=6838** | **mf-ve**  **n=1338** | **mf+ve (%)**  **n=4** | **Total N=1342** | **Nodules**  **-ve**  **N=1339** | **Nodules +ve (%)**  **N=4** | **Total**  **N=1343** |
| **APOD** | 134 (4.0%) | 93 (2.8%) | 227 | 195 (3.6%) | 38 (2.6%)) | 233 | 5 (0.4%) | 0 | 5 | 5 (0.4%) | 0 | 5 |
| **CPOD** | 48  (1.4%) | 104 (3.2%) | 152 | 96 (1.8%) | 59 (4.1%) | 155 | 11  (0.8%) | 0 | 11 | 11 (0.8%) | 0 | 11 |
| **LOD** | 2 (0.1%) | 3 (0.1%) | 5 | 1 (0.02%) | 4 (0.3%) | 5 | 0 | 0 | 0 | 0 | 0 | 0 |
| **Atrophy** (Individuals aged <50 yrs) | 72/3152 (2.3%) | 287/2746 (10.5%) | 359 | 196/4949 (4.0%) | 171/1073 (15.9%) | 367 | 46/1209 (3.8%) | 1/4 (25%) | 47 | 46/1212 (3.8%) | 1/2 (50%) | 47 |
| **Atrophy** (Individuals ≥ 50 yrs) | 118/215 (54.9%) | 353/530 (66.6%) | 471 | 230/403 (57.1%) | 257/365 (70.4%) | 487 | 69/129 (53.5%) | 0 | 69 | 67/127 (52.8%) | 2/2 (100%)) | 69 |
| **Depigmentation** | 31 (0.9%) | 178 (5.4%) | 209 | 98 (1.8%) | 118 (8.2%) | 216 | 3 (0.2%) | 0 | 3 | 4 (0.3%) | 0 | 4 |
| **Hanging Groin** | 15 (0.5%) | 80 (2.4%) | 95 | 33 (0.6%) | 62 (4.3%) | 95 | 1 (0.1%) | 0 | 1 | 1 (0.1%) | 0 | 1 |
| **Nodules** | 216 (6.4%) | 1182 (36.1%) | 1398 |  |  |  | 4 (0.3%) | 0 | 4 |  |  |  |
| **Itching with clinically normal skin** | 380 (11.3%) | 246 (7.5%) | 626 | 645 (12.1%) | 0 | 645 | 25 (1.9%) | 0 | 25 | 25 (1.9%) | 0 | 25 |

* Number (%) skin-snipped: Mesoendemic villages = 6,643/6790 (97.8%); Nonenendemic villages = 1,342/1343 (99.9%)
