# Supplementary material for: Pre-control relationship of onchocercal skin disease with onchocercal infection in Guinea Savanna, Northern Nigeria
Source: PLoS Negl Trop Dis. 2017 Mar 29;11(3):e0005489. doi: 10.1371/journal.pntd.0005489 (PMC5386293; doi:10.1371/journal.pntd.0005489)
Supplement: S3 Table — (DOCX) [file pntd.0005489.s006.docx]

**S3 Table Prevalences of non-onchcercal skin disease in endemic and nonendemic villages (multivariate analysis correcting for age and gender).**

| **Skin condition** | **Endemic villages N=6790** | | **Nonendemic villages N=1343** | | **Univariable OR^a^ (95% CI)** | **P value** |
| --- | --- | --- | --- | --- | --- | --- |
|  | **n** | **% (95% CI)** | **n** | **% (95% CI)** |  |  |
| **Acne** | 970 | 14.3 (9.4-19.2) | 381 | 28.4 (5.0-51.7) | 0.376 (0.238-0.593) | <0.001 |
| **Pyoderma** | 622 | 9.2 (7.3-11.0) | 44 | 3.3 (2.8-3.8) | 3.118 (2.462-3.950) | <0.001 |
| **Scabies** | 298 | 4.4 (1.4-7.4) | 14 | 1.0 (0.0-4.5) | 4.515 (1.992-10.230) | =0.001 |
| **Pityriasis versicolor** | 234 | 3.4 (2.4-4.5) | 78 | 5.8 (0.0-20.6) | 0.597 (0.395-0.901) | =0.016 |
| **Miliaria** | 203 | 3.0 (0.0-4.6) | 7 | 0.5 (0.0-6.4) | 6.160 (1.502-25.26) | =0.013 |
| **Dermatophyte infection** | 96 | 1.4 (0.8-2.1) | 33 | 2.5 (1.1-3.8) | 0.571 (0.359-0.908) | =0.019 |
| **Insect bites** | 29 | 0.4 (0.3-0.6) | 16 | 1.2 (0.0-2.9) | 0.360 (0.236-0.549) | <0.001 |
| **Other skin diseases** | 2,350 | 34.6 (24.5-44.7) | 669 | 49.8 (14.2-85.4) | 0.522 (0.325-0.837) | =0.008 |
| **Any non-OSD** | 3,644 | 53.7 (40.4-67.0) | 928 | 69.1 (33.0-100) | 0.521 (0.296-0.918) | =0.025 |
